# Relationship between the concentration of ergothioneine in plasma and the likelihood of developing pre-eclampsia

**DOI:** 10.1042/BSR20230160

**Published:** 2023-07-06

**Authors:** Louise C. Kenny, on behalf of the SCOPE Consortium, Leslie W. Brown, Paloma Ortea, Robin Tuytten, Douglas B. Kell

**Affiliations:** 1 Department of Women’s and Children’s Health, Faculty of Health and Life Sciences, University of Liverpool, Liverpool L7 8TX, U.K.; 2 Metabolomic Diagnostics, Cork, Ireland; 3 Department of Biochemistry and Systems Biology, Institute of Systems, Molecular and Integrative Biology, University of Liverpool, Crown St, Liverpool L69 7BX, U.K.; 4 Novo Nordisk Foundation Centre for Biosustainability, Technical University of Denmark, Kemitorvet 200, 2800 Kgs Lyngby, Denmark

**Keywords:** Ergothioneine, Metabolomics, Oxidative stress, Preeclampsia

## Abstract

Ergothioneine, an antioxidant nutraceutical mainly at present derived from the dietary intake of mushrooms, has been suggested as a preventive for pre-eclampsia (PE). We analysed early pregnancy samples from a cohort of 432 first time mothers as part of the Screening for Endpoints in Pregnancy (SCOPE, European branch) project to determine the concentration of ergothioneine in their plasma. There was a weak association between the ergothioneine levels and maternal age but none for BMI. Of these 432 women, 97 went on to develop pre-term (23) or term (74) PE. If a threshold was set at the 90th percentile of the reference range in the control population (≥462 ng/ml), only one of these 97 women (1%) developed PE, versus 96/397 (24.2%) whose ergothioneine level was below this threshold. One possible interpretation of these findings, consistent with previous experiments in a reduced uterine perfusion model in rats, is that ergothioneine may indeed prove protective against PE in humans. An intervention study of some kind now seems warranted.

## Introduction

Pre-eclampsia (PE) is a multi-system disorder of pregnancy, characterized by gestational hypertension and the new-onset of proteinuria and/or another maternal organ dysfunction [1–3]. It is widely considered to develop in two stages [4,5]: an initial poor placentation [6] followed by oxidative stress and inflammation [7,8]. As with many other chronic, inflammatory diseases [9], there is also substantial evidence for the involvement of a microbial component [10,11]. PE can affect 3–5% of pregnancies worldwide [12], and has the potential to be life-threatening; the only real clinical recourse currently available is the premature delivery of the fetus. Early markers for predicting PE are thus highly desirable.

Although some soluble protein markers such as sFlt1 and PlGF have proven to be of value (e.g. [13,14]), a number of studies have developed the idea that small molecules (i.e. metabolomics [15,16]) have the potential to provide both diagnostic information, and, via mechanistic reasoning, potentially treatments [17–29].

Ergothioneine is an important antioxidant nutraceutical (commonly derived from the dietary intake of mushrooms) [30–35], for which humans have evolved at least one transporter [36,37]. In one large-scale metabolomics study [38], it was by some distance the molecule most associated with the prevention of adverse cardiovascular outcomes. Its levels have also been linked with lowered incidences of cognitive defects [39], and it prevented some of the main symptoms in the rat RUPP model of PE [40]. In view of the above, and its status as a potent antioxidant (and its actions via other unknown mechanisms [41]), it was thus considered plausible to have some utility in the prevention and/or diagnosis of PE in humans [42].

The international Screening for Pregnancy Endpoints (SCOPE) study [25,43–45] (http://scopestudy.net) bio-banked early pregnancy blood samples from a large cohort of first time pregnant women, 4.9% whom developed PE as defined [46] by the International Society of the Study of Hypertension in Pregnancy [43]. Pre-term PE was defined as disease necessitating delivery before 27 weeks’ gestation. The SCOPE study therefore provided the opportunity to assess whether there was any relation between their levels of ergothioneine and the likelihood of developing early or late-onset PE. The present study reports on a secondary analysis of ergothioneine levels in a previously reported case-control study assessing a panel of metabolite biomarker candidates for PE risk assessment at 15 +/-1 week of gestation [25]. Women with the highest level of ergothioneine had a significantly reduced likelihood of developing either preterm or term PE. One interpretation of such data could be that dietary supplementation with ergothioneine in pregnancy may reduce PE risk, and this is obviously now worth testing. A preprint has been lodged (https://www.medrxiv.org/content/10.1101/2022.12.19.22283617v1).

## Results

### Overall summary of the cohort studied

The nested Case-Control study was defined in the SCOPE – Europe cohort as described fully in [25], and the demographics (Table 1 of [25]) are not repeated here. For this work, we leveraged calibration data available in the previous study to estimate the ergothioneine blood levels (ng/ml) in 432 women of whom 335 did not develop PE, 23 suffered preterm PE, and 74 suffered from term PE.

**Table 1:** Frequentist statistical analysis of the three pregnancy outcome classes here considered

Pregnancy outcome	No PE	Preterm PE	Term PE
*N*	335	23	74
*Ergothioneine (ng/ml)*			
*Min – Max*	140–998	149–383	166–497
*Mean (95% CI)*	294 (281–307)	259 (234–294)	276 (259–293)
*Median (95% CI)*	260 (248–271)	268 (230–288)	260 (247–282)
*Standard Deviation*	123	58	73
*25-75^th^ Percentile (IQR)*	211–332	214–290	220–333
*10-90^th^ Percentile*	184–462	183–340	188–382
Normal Distribution (p)*	<0.0001	0.34	0.13

*Shapiro–Wilk test; if *P*< 0.05: Reject Normality; IQR: Interquartile Range; 95% CI: 95% Confidence interval.

### Ergothioneine levels in the study population

**Figure 1:**
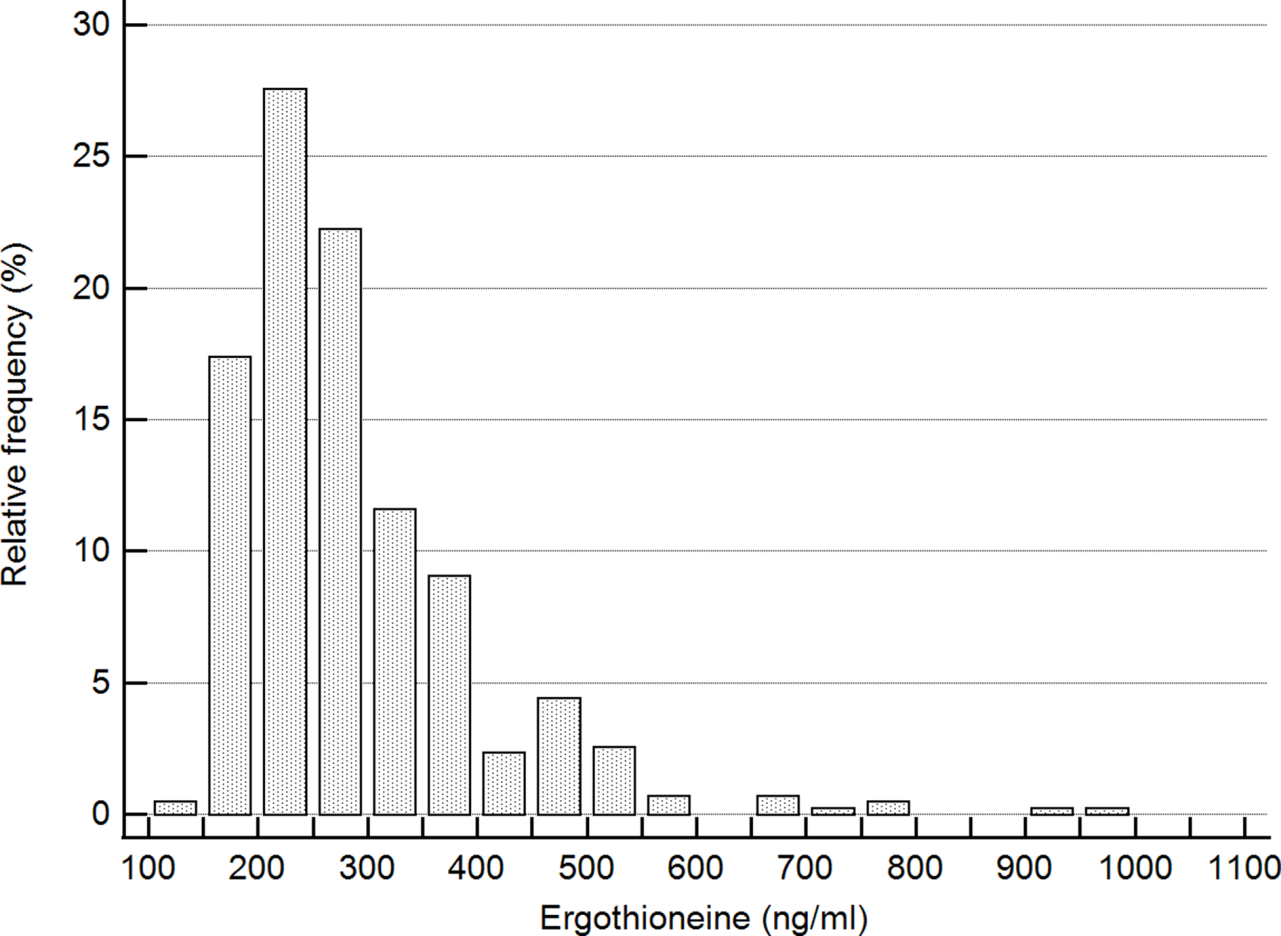
Distribution of ergothioneine levels in the study population

In Figure 1, the distribution of ergothioneine levels in the study-subjects is plotted.

The levels range from ∼140 to 998 ng/ml, with a median level of ∼260 ng/ml. From Figure 1, it is clear that ergothioneine levels are not normally distributed within the study population, but that the long-tailed distribution is skewed to higher concentrations.

**Figure 2:**
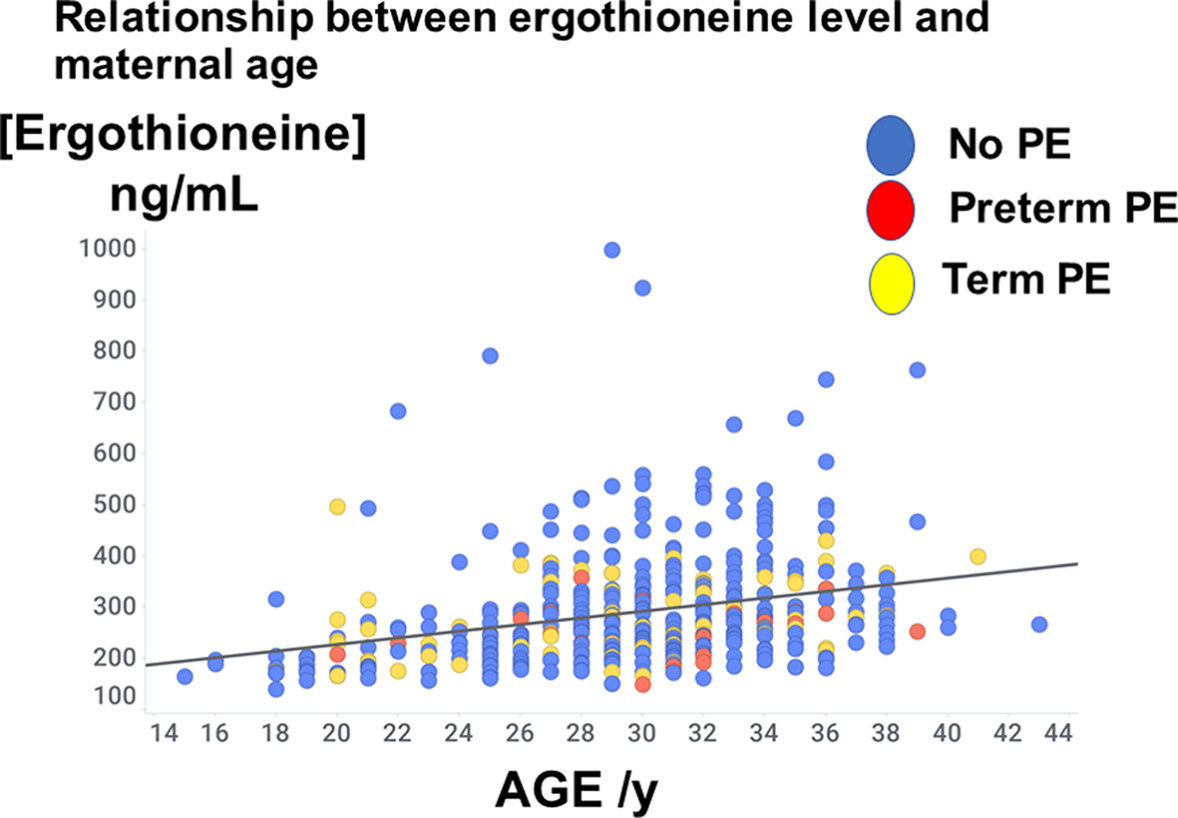
Relationship between ergothioneine level and maternal age

**Figure 3:**
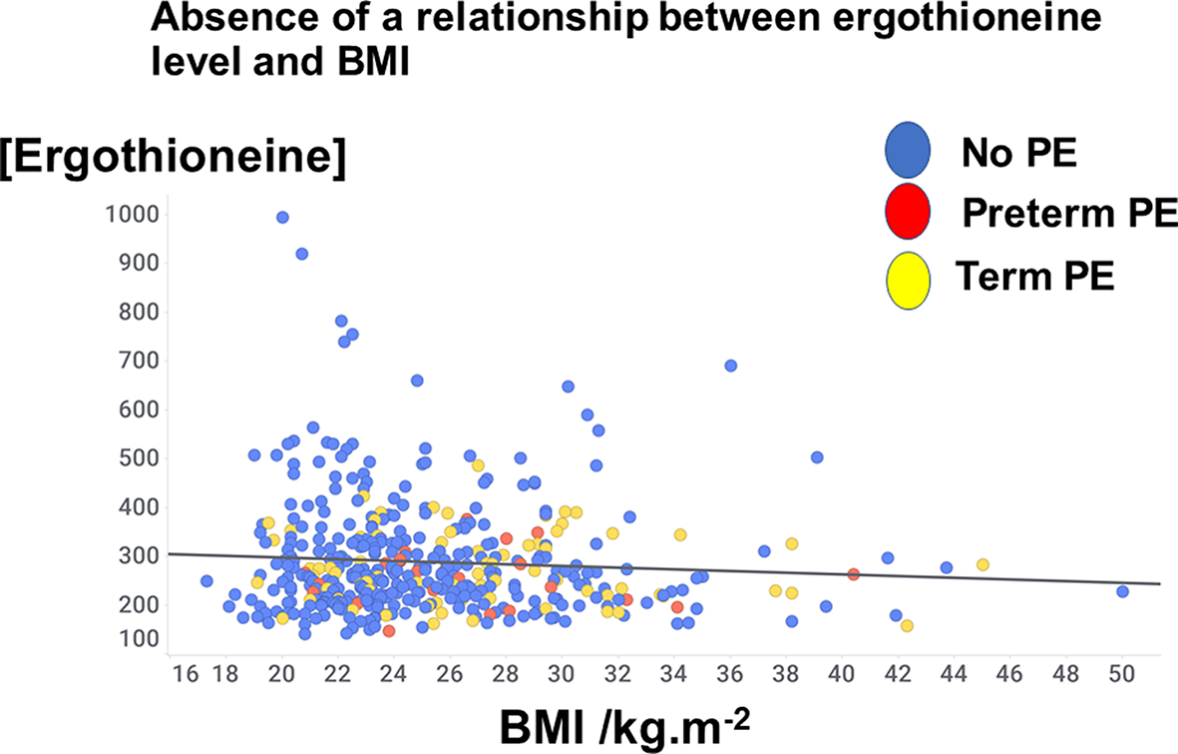
Lack of relationship between ergothioneine level and BMI. Data have been jiggered in the *x*-axis direction for clarity

The distribution of ages may be observed in Figure 2, where it may also be seen that there was a weak yet significant correlation of ergothioneine levels with age (*r*^2^ = 0.08, *P*<0.0001). When the analyses were confined to those with an ergothioneine concentration greater than 462 ng/ml (see below), the ‘correlation’ was negative, with an *r*^2^ value of 0.004; maternal age was not a confounding factor. Similarly, there was no significant relationship (*r* = −0.07, *P*=0.14) between ergothioneine levels and BMI (Figure 3).

### Levels of ergothioneine in control and in preclamptic women

**Figure 4:**
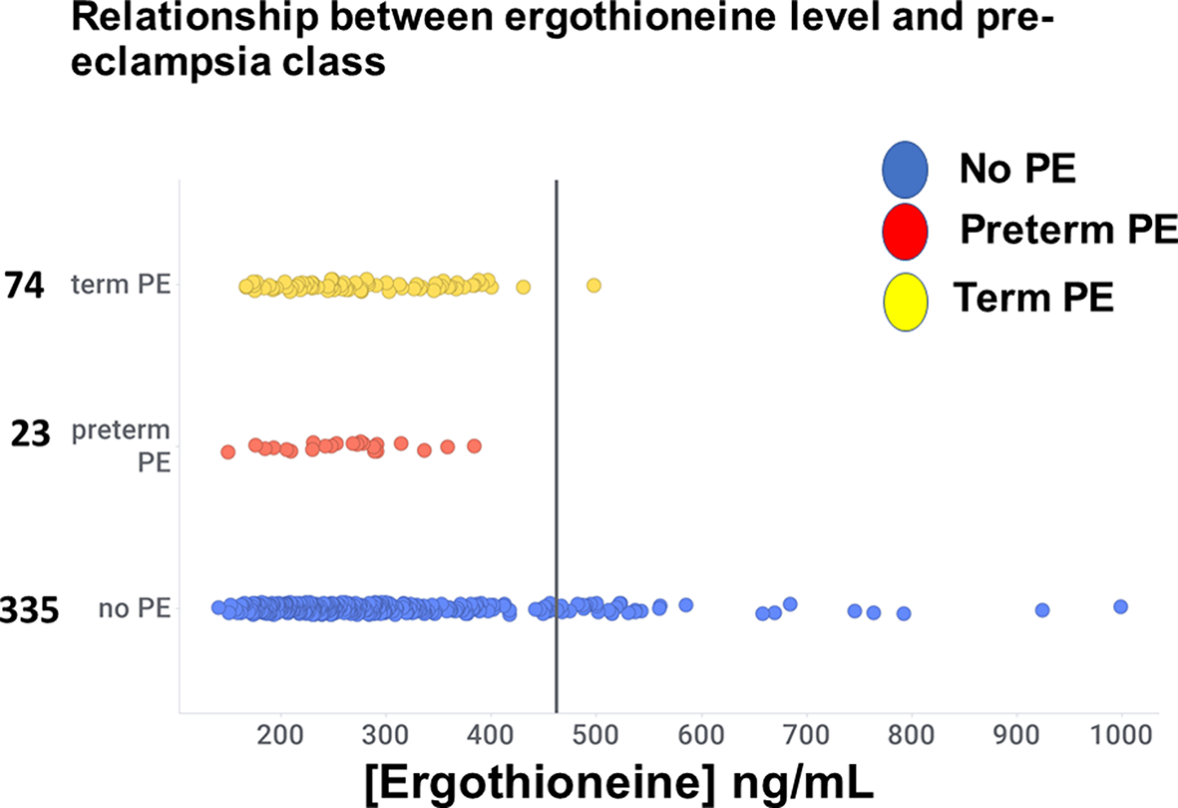
Relationship between ergothioneine level and PE category Data have been jittered in the y-axis direction for clarity

A chief finding here is illustrated in Figure 4, where we show the relationship between the levels of ergothioneine and whether the women concerned developed PE, whether at term or pre-term. Taking a threshold of >462 ng/ml, which is equivalent to the 90th percentile in the control population, we see that only 1/74 developed PE at term, and 0/23 developed PE pre-term, i.e. only 1/97 (1%) when these numbers are combined developed any form of PE.

33/335 (10%) who did not develop PE were also above this threshold. In other words, only a single individual out of 432 examples both had a level of ergothioneine above 462 ng/ml and developed any form of PE.

The ergothioneine levels for those three classes are given in Table 1, where it is clear that without considering the specific distributions in detail the mean and median figures would not have indicated an association between ergothioneine and PE risk. From the distributions as plotted in Figure 4, it can be inferred that within the study population, there is sub-set of study participants who have markedly higher ergothioneine blood levels, giving rise to a bimodal distribution within the study population.

**Figure 5:**
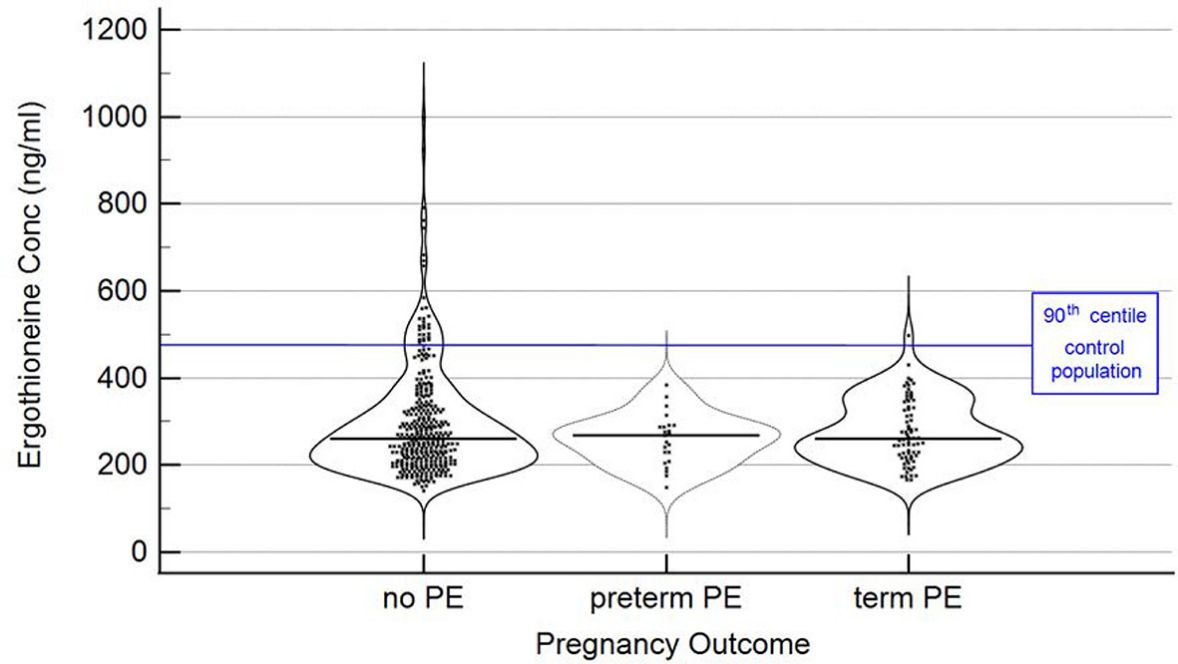
Violin plots based on individual measurements Violin plots based on individual measurements; — median levels; blue line: 90th centile based on ergothioneine distribution in the control population. Data as in Figure 4 with statistical summary in Table 1.

The relationship shown in Figure 4 (i.e. setting a cut-off at the 90th centile) was post-hoc, yet it should be evident that a data-driven, machine learning analysis (to be contrasted with a frequentist statistical approach [47,48]) would have discovered it, much as it did [22] in our first foray into PE metabolomics (using a low-resolution mass spectrometer that—as is still common even with high-resolution instruments [49–52]—could not identify most peaks of interest). We also note that reference-range-based rules are commonly used in clinical diagnostics to identify a population of interest, whereby patients with blood levels of a marker of interest in, e.g., the top or bottom 10% of the reference interval are flagged (Figure 5).

Using the above 90th centile as a threshold to identify a population of interest, which is non-arbitrary and a common practice with nutraceuticals (e.g. [53–55]), the following Odds Ratio for developing PE is found in this group; OR = 0.095 with 95% confidence interval (0.0129–0.706) and significance level *P*<0.02.

## Discussion

Stimulated by the recognition that ergothioneine might be a potentially useful therapeutic in PE [40,42], and that it is certainly a potent (and safe [56]) antioxidant nutraceutical [30,32–34,57–64], we assessed its concentration in early pregnancy in a representative sample of first-time pregnant women who participated in the European branch of the SCOPE project. Certainly the range of concentrations observed was substantial, from an estimated ∼140 ng/ml up to ∼1 μg/ml (Figure 3). Within the study population, the median levels were found to be non-differential between women who developed preterm-, term- PE or women who did not develop PE later in pregnancy. However, within the women who had ergothioneine levels ≥ the 90th percentile of the reference population, i.e., women who did not develop PE, only one study participant developed PE.

The levels of ergothioneine reflect both intake (especially from mushrooms [65,66]) and the activity of the various ergothioneine transporters [36,37,67–69], and neither of these were either known or controlled. Thus, as with a related study on cardiovascular event incidence, where ergothioneine was strongly (indeed the molecule most strongly) associated with more favourable outcomes in terms of morbidity and mortality [38], this was a purely observational study. Ergothioneine was also the metabolite associated with the lowest hazard ratio for all-cause mortality [70], and had the third highest loading in a signature collection of healthy metabolites [71]. Mushroom consumption is also strongly associated with a lowering of all-cause mortality [72] and of the incidence of mild cognitive impairment [73]. Consequently, since certain aspects of the PE syndrome share hallmarks of vascular disease [74–76], this study adds weight to the idea that it might be a useful nutraceutical in the prevention of (cardio)vascular diseases more generally.

## Materials and methods

Ergothioneine was one of the compounds analysed with multiplex targeted liquid chromatography-tandem mass spectrometry assay for PE biomarker candidates as detailed in [25]. In the latter study, biomarker levels were expressed as relative concentrations whereby for any sample the target metabolite read-out was divided by the read-out as obtained from a stable-isotope labelled metabolite internal standard spiked in equal amounts across all samples. Deuterated ergothioneine (D9) served as the stable-isotope labelled metabolite internal standard for ergothioneine quantification.

For this secondary data analysis, the relative ergothioneine concentrations were converted in estimated blood levels (ng/ml) using the calibrators co-analysed with the patient samples [25].

In brief, calibration curves were generated by means of firstly fortifying a pooled plasma (Technopath plasma with 2% K2-EDTA anticoagulant Lot PF-05171, Technopath, Ireland) with the metabolites of interest and then serially diluting the fortified sample with PBS/BSA buffer (0.01M phosphate buffer and 0.5% Albumin). The metabolites levels for the fortified matrix as well as the concentration span were estimated based on preliminary evaluations. This led to the creation of an 8-point calibration curve for all metabolites spanning a ∼20-fold dynamic range (CAL1; relative level = 100 to CAL8, relative level = 5.83). In a separate experiment the levels of ergothioneine in the pooled plasma were determined by means of standard addition [77], yielding an estimated level of 138 ng/ml. Using this information, the ergothioneine calibration range expressed in ng/ml is easily derived (174 ng/ml [CAL8] – 752 ng/ml [CAL1]). It is noted that said calibration range covers 93.5% of all patient samples assessed; moreover, we typically find that the linear range extends beyond the set calibration range for the metabolites assessed. Verification that the ergothioneine calibrator curves effectively mitigated technical variability followed the estimation of imprecision from the analysis of 73 duplicate patient samples (independently prepared and randomly distributed across study batches) using the method of Hyslop and White [78], returning a satisfactory Coefficient of Variation (%) = 11.5%.

## Conclusions

The very striking observation that, in this cohort, only 1 individual out of 97 (1%) women with an ergothioneine level above 462 ng/ml manifested PE, whereas in the total cohort 97/432 (22.5%) did, demands an explanation. The easiest one is that this molecule is significantly protective towards (and may be consumed during) the development of PE. It is worth noting that, as well as its occurrence in all known culinary mushrooms, ergothioneine is an available nutritional supplement, whose safety has been well established [56,79–82]. Such an analysis will best be done via a randomized controlled trial.

## Supplementary Material

Supplementary Table S1

## Data Availability

The dataset is provided as an Excel sheet in Supplementary Information.

## Open Access

Open access for this article was enabled by the participation of University of Liverpool in an all-inclusive *Read & Publish* agreement with Portland Press and the Biochemical Society under a transformative agreement with JISC.

## Abbreviations

IQR: interquartile range
PE: pre-eclampsia
SCOPE: Screening for Endpoints in Pregnancy
